# Using Facebook data to predict the 2016 U.S. presidential election

**DOI:** 10.1371/journal.pone.0253560

**Published:** 2021-12-01

**Authors:** Keng-Chi Chang, Chun-Fang Chiang, Ming-Jen Lin

**Affiliations:** 1 Department of Political Science, University of California, San Diego, La Jolla, California, United States of America; 2 Department of Economics, National Taiwan University, Taipei, Taiwan; Georgia State University, UNITED STATES

## Abstract

We use 19 billion likes on the posts of top 2000 U.S. fan pages on Facebook from 2015 to 2016 to measure the dynamic ideological positions for politicians, news outlets, and users at the national and state levels. We then use these measures to derive support rates for 2016 presidential candidates in all 50 states, to predict the election, and to compare them with state-level polls and actual vote shares. We find that: (1) Assuming that users vote for candidates closer to their own ideological positions, support rates calculated using Facebook predict that Trump will win the electoral college vote while Clinton will win the popular vote. (2) State-level Facebook support rates track state-level polling averages and pass the cointegration test, showing two time series share similar trends. (3) Compared with actual vote shares, polls generally have smaller margin of errors, but polls also often overestimate Clinton’s support in right-leaning states. Overall, we provide a method to forecast elections at low cost, in real time, and based on passively revealed preference and little researcher discretion.

## Introduction

Scholars have used social media data to measure elite and mass ideology [1, 2], but few efforts have been made to compare measures from social media with polls and to forecast elections using social media data. To our knowledge, most related work to date mostly focus on count of mentions, sentiments of texts, or by extracting large numbers of textual features [3]. None of which are based on any theoretical foundations and involves large amount of training data and researcher discretion.

In this paper, we specify a large group of possible ideological-related fan pages and explore one of the most common actions on Facebook: *likes*. By assuming that users are more likely to *like* the posts from fan pages that are closer to their own ideological position, we are able to place politicians, news outlets, interest groups, and ordinary citizens on the same ideological spectrum. This passive measure does not rely on users’ self-reporting, can be in almost real time, and can trace certain users repeatedly. Additionally, since we are looking at the *liking of posts* (as opposed to *following of pages* in [1]), this measure adds the whole universe of time and post content dimensions to our analysis.

We use this measure to derive support rates and predict the 2016 US Presidential election. First, we make assumptions about the user’s location at state-levels (by their likes on Senators, member of the House of Representatives, and Governors) and combine the user’s and the candidate’s ideological positions so that we can calculate support rates (share of users closer to each candidate in that state in terms of ideology) for two major Presidential candidates in all 50 states based on the standard spatial models [4]. Given the limited information we know about the users and the minimal assumptions we made, the calculated support rate before the election predicts that Trump would win 293 electoral college votes while Clinton would win the popular vote.

Next, we compare the dynamic support rates in each states with the state-level polling averages calculated by FiveThirtyEight.com. A cointegration test confirms the time trends between Facebook support rates and polling averages share similar trends. Furthermore, by comparing Facebook support rates and polling averages with actual vote shares, we find that polling averages systematically overestimate Clinton’s support in right-leaning states, while Facebook support rates often overestimate those of Trump.

This paper proceeds as follows. Section briefly reviews the literature. Section describes the data and the procedure to obtain our measure of ideology using Facebook data. Section reports how we use this measure to predict elections and to compare with state polls and actual vote shares. Section discusses some strengths and weaknesses of using Facebook data and finally concludes.

## Related literatures

### Ideal points of political elites

There is a vast literature on estimating the ideal point of politicians. [5] builds their foundational work on supposing legislators would vote for roll-calls that are closer to their own ideal point. [6] extends the procedure into a Bayesian setting that is more flexible to incorporate other information (priors) and can be estimated using Markov Chain Monte Carlo (MCMC) simulations through maximizing the posterior distribution. Other than likelihood-based methods, [7] uses a form of dimension reduction to estimate legislative preference that lowers computational costs and achieves similar results presented in DW-Nominate method.

A limitation of this line of research is that we cannot apply it to people outside Congress. [8] provides a creative breakthrough by making use of campaign finance data to jointly estimate the ideological positions of campaign finance contributors and receivers.

### Media bias

Political information is often provided by news outlets. Several studies have provided evidence that most media have their own bias in processing political information.

For example, [9] links media and politicians by counting the times each news outlet cites a particular think tank and compare it to the times the members of Congress cite those think tanks. [10] compares the phrase usage of newspapers to that of congressperson on the Congressional Record. [11] links media and the public by looking at browsing records of news outlet websites and visitors’ self-reported ideology.

### Political ideology on social media

[1, 2] are two main contributions to estimating ideological scores using social network data. Assuming that people tend to follow politicians closer to their own unobserved ideological position, [2] uses Twitter data and [1] uses Facebook data to estimate a joint ideology score for politicians and the general public.

However, they generally only consider the links between users and politicians, neglecting other important political participants, such as news outlets and interest groups. Additionally, following or liking fan pages is usually a one-shot action. Using only following data on Facebook is perhaps a waste of information, since data on the liking of posts on fan pages is not only publicly available, it also may provide time and post content level dimensions to provide a richer understanding of human behavior.

### Ideology of the general public

Ideology measurements of individuals are generally evaluated in surveys. Researchers usually ask respondents to place themselves on a 7-point liberal-conservative scale (see General Social Surveys [12] and American National Election Study [13]).

This method, though convenient and straightforward, has some potential problems. For example, respondents may interpret the questions differently [14], or there may be certain social pressure for respondents to respond in a certain way [15, 16]. Additionally, it also does not account for the multidimensional nature of ideology if separate questions for economic, moral, or other social or policy issues are not presented. Using a revealed preference approach through people’s behavior on social media may be a good complement to our current understanding of mass ideology through surveys.

### Social media and election prediction

Most existing work trying to predict polls and elections are based on text data within Twitter. Some early attempts were made in the artificial intelligence community [17–19]. [17] trains a model based on party mentions. [18] trains a model based on text and graph structures, such link centrality and party-speech centrality. [19] trains a ensemble based on campaign-relevant Twitter messages, claiming that volume-based and sentiment-based alternatives perform worse on predictions. Perhaps the most recent and important attempt for the social science community is [3], which extracts textual features from Twitter to train and predict state-level polls.

Our study is fundamentally different from these existing methods in at least two ways. First, there is no training phase in our approach. We adopt state of the art methods to scale the ideological positions based purely on Facebook data and use that directly to predict the outcomes in polls and the actual election results. One can easily think of other ways to train models by combining information from both Facebook and polls and use that to predict election outcomes, but this leaves huge discretion for the researchers to cherry-pick the result that generates the best prediction.

Second, none of the methods above involves any social science theories about how people vote. We adopt the most widely-used model of voting in political science and economics to inform how we can make election predictions. More complicated models might increase the accuracy of the prediction, but it is worth documenting that simple and classical models can already travel a long way.

## Facebook ideology measure

### Facebook data

Facebook provides fan page data and user’s behavior on fan pages through their Graph API. The Graph API mainly includes posts of the fan pages and the users’ reaction to these posts.

To collect a set of pages related to political ideology, we focus on fan pages relevant to the 2016 US Presidential election but we do not want to handpick the pages to prevent possible selection bias. Specifically, we combine two sets of pages into our main sample.

First, we select fan pages that mentioned two major presidential candidates, Donald J. Trump and Hillary Clinton, in August 2016. We calculate the total number of likes, comments, and shares of candidate-related posts in these pages, and weight them by factors 1:7:14, respectively, to determine which pages to include. Since no one knows the exact algorithm Facebook adopted to calculate EdgeRank, the score that determines post visibility, we use the weights proposed by [20]. Additionally, changing the weights does not change the pool of pages much. We end up with the top 1000 election-related pages that include all major news outlets, presidential candidates, and policy interest groups.

Second, we include all fan pages of current national politicians, including members of and candidates for the Senate, the House, and past and present Governors. Many politicians own two pages, one official page (ex. U.S. Senator Bernie Sanders), and one personal page (ex. Bernie Sanders). We include all of them and use the page that generates more posts to represent the politician when necessary. We end up with a total of 1475 politician fan pages (with 1225 pages having posted in 2015 or 2016).

We collect all the posts and reactions to these posts from these two sets of political-related pages from January 2015 to December 2016.

In some cases, we restrict our sample to what we define as *US political users*, which are the users that have ever reacted to at least one post on the 1475 national politician fan pages in 2015 and 2016. Many U.S. news outlets and some politicians are also well-known globally, but what we would like to know is the ideological positions of these players in U.S. citizens’ eyes. Restricting the sample provides a better approximation for what we want to study, given that we know nothing about a user other than their ID.

**Fig 1:**
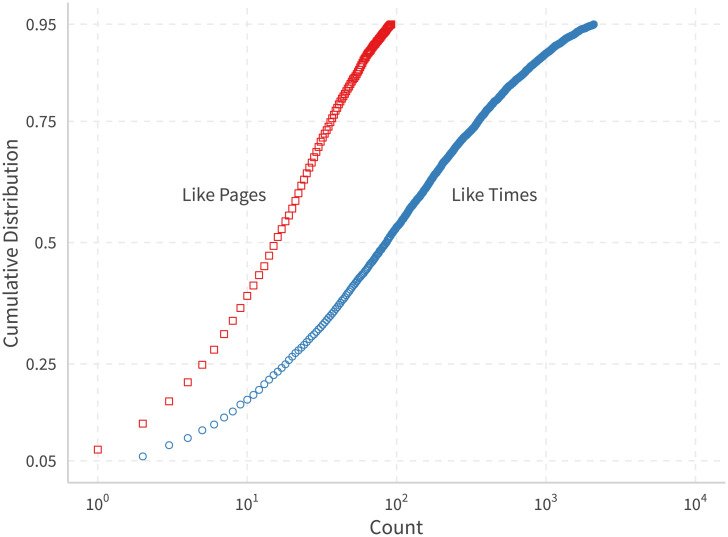
Distribution of pages and post per user likes. x-axis is log scaled.

Table 1 gives a brief summary of our main sample. Fig 1 shows the cumulative distribution of the number of pages and likes (of posts) by US political users on these pages. The distribution is quite light-tailed–with 50% of users liking only 16 different pages and 86 different posts, and 10% of users liking more than 68 pages and 1176 posts.

**Table 1:** Data summary (main sample).

Time Period	2015-01-01 to 2016-11-30
Total Reactions	19,085,783,534
US Political User Likes	16,180,488,916
Total Users	366,840,068
US Political Users	29,412,610
Total Posts	24,788,093
Total Pages	2132
Politician	1225
News Outlets	560
Political Groups	211
Other Public Figures	93
Others	43

*Notes*: US political users are defined by any user that at least reacted to any national politicians’ (Sen, Rep, Gov) post once in 2015 and 2016.

### Scale ideology through dimension reduction

We adopt a similar procedure proposed by [1]. Since what we analyze is the reaction to posts, we define *fans* of a page to be U.S. users that *like* at least one post in that page in a given period of time. We do not include other reactions (love, haha, wow, sad, and angry) to make interpretations easier. Then, we are able to construct an affiliation matrix (see Table 2 for an example). The diagonal elements of this matrix are the numbers of unique fans on each page. The off-diagonal elements are the numbers of shared fans between pages. The time period selected in this example includes posts from 2015-01-01 to 2016-11-07. We can observe that there are large differences between shared fans among different pages.

**Table 2:** Affiliation matrix (part).

	Trump	FoxNews	TeaParty	Clinton	CNN	NYTimes
Trump	2,243,216	1,078,513	128,225	32,731	120,963	25,842
FoxNews	1,078,513	2,449,174	148,016	87,084	186,850	63,401
TeaParty	128,225	148,016	242,089	1528	10,738	2,162
Clinton	32,731	87,084	1528	1,768,980	351,210	367,021
CNN	120,963	186,850	10,738	351,210	1,201,156	216,163
NYTimes	25,842	63,401	2162	367,021	216,163	986,613

*Notes*: Diagonal numbers are unique US political users like at least one post of each pages, off-diagonal numbers are shared unique US political users at least one post in both pages. Data ranges from 2015-01-01 to 2016-11-07. US political users are defined by any user that at least reacted to any national politicians’ (Sen, Rep, Gov) post once in 2015 and 2016.

We then transform the affiliation matrix to an agreement matrix in order to extract meaningful features from shared fan data (see Table 3 for an example). For each element in affiliation matrix $\vec{A}$, we compute *g*_*ij*_ = *a*_*ij*_/*a*_*ii*_ to get agreement matrix $\vec{G}$. For example, 0.48 is the number of shared fans between Trump and Fox News divided by the total number of Trump fans, while 0.44 is the number of shared fans between Trump and Fox News divided by the total number of Fox fans.

**Table 3:** Agreement matrix (part).

	Trump	FoxNews	TeaParty	Clinton	CNN	NYTimes
Trump	1.00	0.48	0.06	0.01	0.05	0.01
FoxNews	0.44	1.00	0.06	0.04	0.08	0.03
TeaParty	0.53	0.61	1.00	0.01	0.04	0.01
Clinton	0.02	0.05	0.00	1.00	0.20	0.21
CNN	0.10	0.16	0.01	0.29	1.00	0.18
NYTimes	0.03	0.06	0.00	0.37	0.22	1.00

*Notes*: For each row in the affiliation matrix, we divide each element by the diagonal element to get agreement matrix. So the numbers in each row are the proportions of shared fans between that page and the pages in each column. Data ranges from 2015-01-01 to 2016-11-07.

This transformation is meaningful; therefore, we can interpret each row as observations and each column as features, with ratios describing the degree to which each observation possesses those features. For instance, Trump page is 100% similar to Trump feature, 48% similar to Fox News feature, and 1% similar to Clinton feature, since the denominators are all the number of Trump fans.

After getting the agreement matrix, we run a Principal Component Analysis (PCA) on the agreement matrix. This is identical to using SVD if one standardizes the data before starting the decomposition. If $\vec{X}$ is a centered data matrix so it has zero sample mean in each column, the empirical covariance matrix is thus $\vec{C}=n^{-1}{\vec{X}}^{⊺}\vec{X}$. What PCA does is to diagonalize $\vec{C}$ such that $\vec{C}=\vec{V}\vec{D}{\vec{V}}^{⊺}$. The principal components are the projection of the data on the eigenvectors, which are columns of $\vec{X}\vec{V}$. If we employ SVD on $\vec{X}$ such that $\vec{X}=\vec{U}\vec{S}{\vec{V}}^{⊺}$, then the Eckart-Young Theorem [21] says that the nearest possible matrix of rank *k* to $\vec{X}$ is ${\vec{U}}_{k}{\vec{S}}_{k}{\vec{V}}_{k}^{⊺}$, which is basically projecting the first *k* principal components ${\vec{U}}_{k}{\vec{S}}_{k}$ back to the original space. We can also derive that $\vec{U}\vec{S}=\vec{X}{\left({\vec{V}}^{⊺}\right)}^{-1}=\vec{X}\vec{V}$, which are the principal components, since $\vec{V}{\vec{V}}^{⊺}=\vec{I}$ holds in spectral decomposition. The principal axes are linear combinations of the original features. The first principal axis points out the direction that preserves the largest variation in the original data. The first principal component (PC1) projects the original data (agreement matrix) on the first principal axis, which we interpret as the ideology scores of fan pages, which reduces the dimension of the original data from thousands to one. We scale the ideology scores to have mean zero and standard deviation one. We also multiply all scores by -1 when necessary in order to be consistent with the traditional left-right interpretation.

**Fig 2:**
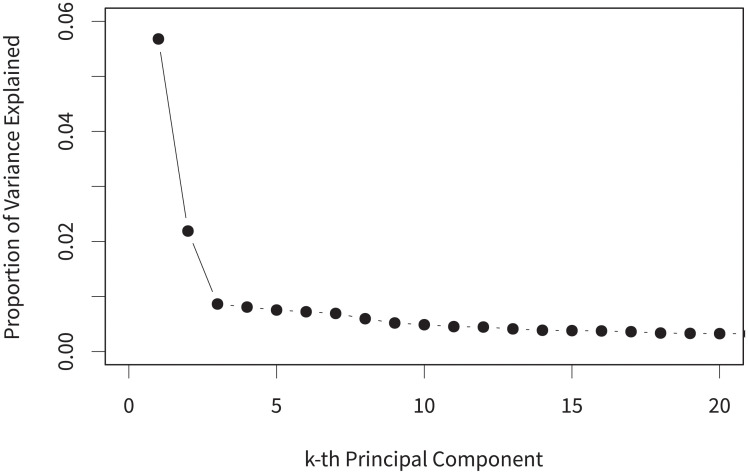
Scree plot for principal component analysis.

Fig 2 presents the scree plot. We can see that proportion of the variation explained for the kth principal component decreases dramatically. This provides evidence that considering the first dimension (the first principal component) may be sufficient for us if we want to focus on the traditional liberal-conservative one-dimensional divide.

### Measures for fan pages

**Fig 3:**
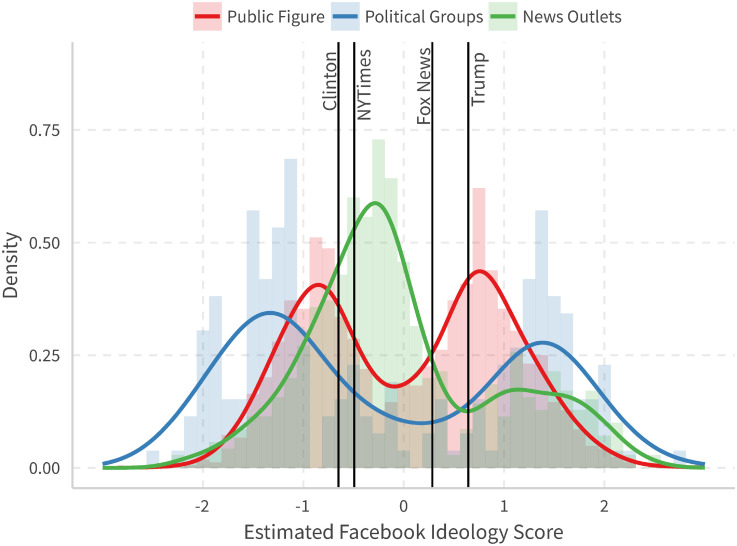
Histogram and density for different page types.

We group the pages into three major categories: news outlets, public figures (including politicians and journalists), and political groups (including parties and policy interest groups). Fig 3 gives the distribution of different page types, using data from 2015-01-01 to 2016-11-07. We also annotate some reference points, such as Trump, Clinton, Fox News, and the New York Times, to give more context to the distribution.

We can observe that news outlets mainly have one mode and public figures and political groups have two modes, while the latter is more dispersed. This is consistent with the roles of these political actors: media serves the general public and interest groups serve politicians. Additionally, note that we can see most media pages are in the center (though slightly left-leaning), though there are also a number of pages that cluster on the right.

**Fig 4:**
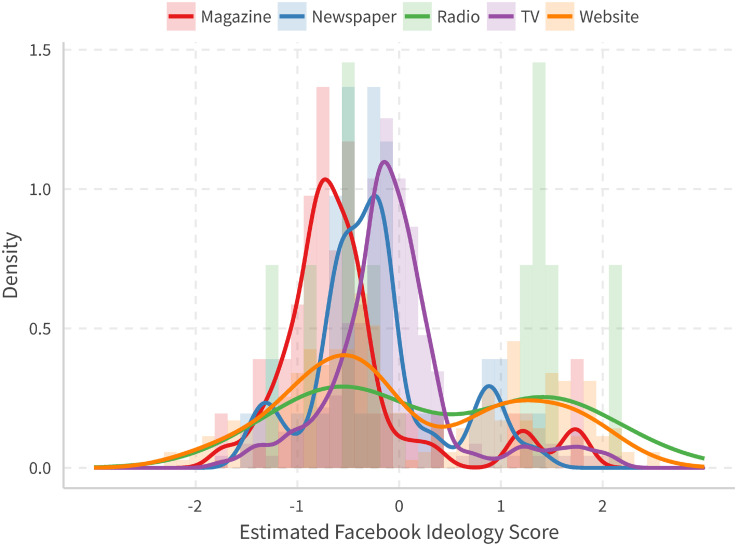
Histogram and density for different media page types.

We can also group media pages into categories. Fig 4 shows the result. One can observe that TV, newspapers, and magazines are quite centered (while more left-leaning accordingly), although radio and website news is more dispersed. Supporting information gives other density plots and annotates some notable pages. For example, S4 Fig shows all the major parties in the US, with Democratic, Green, Libertarian, Republican, and Tea Party from left to right. Most media pages replicate recent studies in media bias, such as [9, 11, and 22].

#### Validations for political ideal points

**Fig 5:**
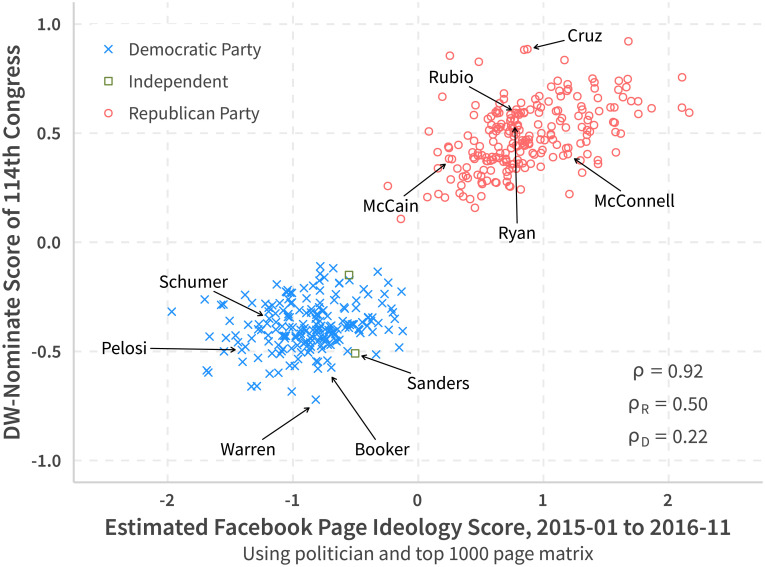
DW-nominate vs. FB estimate (114 Congress).

To validate that our measure captures the liberal-conservative divide, we compare our result with the traditional ideological measure for US politicians: DW-Nominate Score. Fig 5 shows this scatter plot using data for the 114th Congress (2015–2017). Many politicians own multiple fan pages. Here we only use the page that produces more post to represent that politician. Like with DW-Nominate scores, our estimate clearly separates politicians into two groups. The overall correlation between the two measurements is high (0.92), although the correlation inside the Democratic Party is relatively low (0.22).

Nevertheless, if we use only politician pages to form a matrix and calculate ideological positions (method used in [1]), we will get a lower correlation for Democrats (0.15). Additionally, we will get an even lower correlation (0.09) if we use that procedure to forecast the ideology scale in the 115th Congress. This suggests that adding other political-related pages does not confuse, but rather intensifies, our ability to recover people’s perceptions on the hidden political spectrum.

#### Validation for media slants

We can also validate our measure with the degree of media slants. Most existing findings on media slant consider very limited numbers of media, which makes it difficult to perform meaningful comparisons, but the big picture they provide for the major news outlets are largely the same: the New York Times and the Washington Post are to the left, and ABC News, USA Today, and the Wall Street Journal are considered centrist, while Fox News almost monopolizes the major news market of the right.

In this study, we present a similar and yet more interesting validation. Define users to be *Republican-affiliated* if their likes in all politicians are more of Republican politicians (compared with other major parties). We can then compute the *share* of Republican-affiliated users of each news outlet. To remove potential bias created by active users and to be consistent with other papers (such as [11]), we only count a user once per day if they like more than one post of that fan page on that day. We then sum all this kind of so-called *daily* users across day to compute an average share. We use data from 2015-01-01 to 2017-03-31.

**Fig 6:**
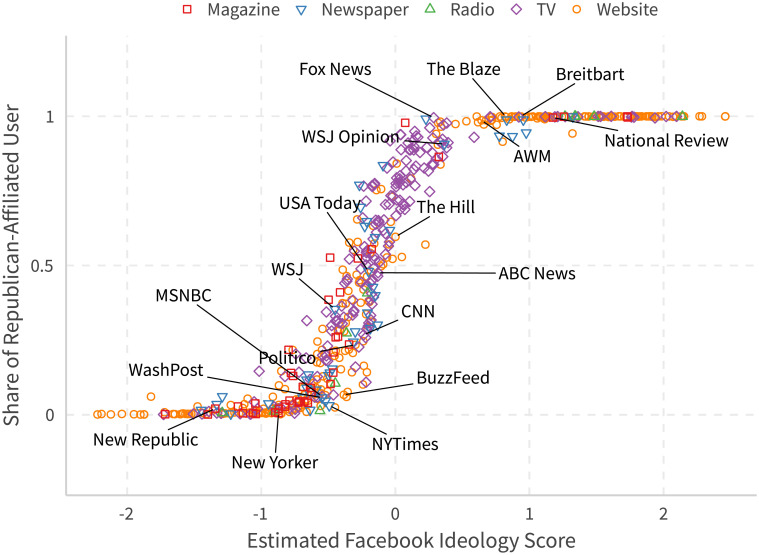
Validation of media slant. A user is *Republican-affiliated* if their likes in all politicians are more of Republicans. We only count user once a day on a page if they like more than one post on that day on that page. We then sum all this kind of daily users up across each day. Data ranges from 2015-01-01 to 2017-03-31.

Fig 6 shows the just mentioned measure against our Facebook Estimate. Our estimate not only replicates both previous studies and the alternative measure, we can see that there are still quite a number of pages that are on both ends of the spectrum (with either almost all or no Republican users; this also highlights a shortcoming of this alternative straightforward measure), and many of them are still quite popular.

This finding indicates one of the strengths of our method. Many studies of media slant have to rely on a predefined pool of news outlets or a choice in surveys. This approach may subject the outlests to some sort of bias imposed implicitly by the researchers since most individuals have a limited knowledge of what others are seeing. Our evidence, not from a presumed pool, shows that there are quite a number of sizable right-wing news sources other than Fox News, but this is still consistent with findings by [9], which suggests a liberal bias for almost all major news outlets.

### Measures for users

After calculating the ideological scales for the pages, we calculate each user’s ideology by averaging the ideologies of the pages the user liked (on their posts). Whether the like times in each pages are taken into account (hence a weighted mean) or median instead of mean is used as a measure does not substantially change the result (correlation > 0.97).

We guess the geographical information (states) of the user by their maximum likes of national politicians (e.g., if one likes more politicians from Texas, one is more likely to be a Texas-related user) and weight them by the 2016 population in each state [23] if that state is overrepresented in our sample relative to the population.

Little is known about the ideological compositions of users on Facebook. [1] mentions that Facebook users are relatively young, white, educated, female, and liberal. However, a caveat is that these conclusions are from data in 2012 when the social media giant was still in its early stage. On the other hand, a recent survey [24] indicates that 26% of Republicans and 25% of Democrats follow public figures.

**Fig 7:**
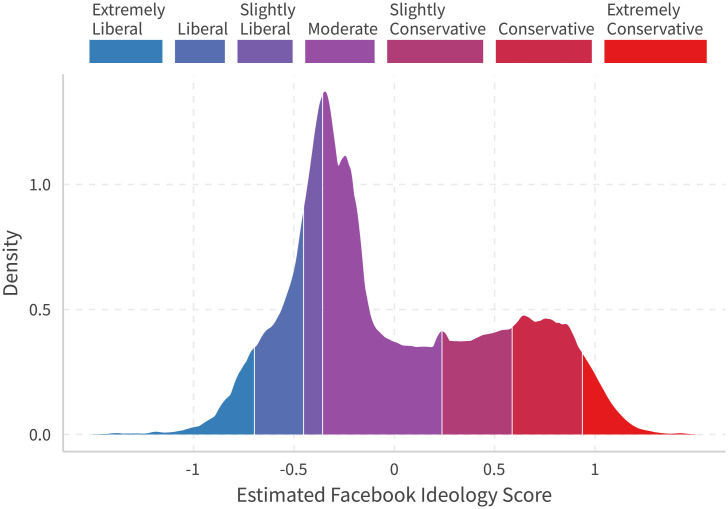
Density for all US users and self-report ideology shares in GSS. Colors represent matching densities with self-reported ideology shares in 2016 General Social Surveys [12]. US users are defined by any user that at least reacted to any national politicians’ (Sen, Rep, Gov) post once in 2015 and 2016, and we guess user’s location by the maximum national politician they liked in that state. We remove a huge jump created by users only like one and only one page: Arnold Schwarzenegger. We then sample users by 2016 population in each state [23] if that state is overrepresented in our sample relative to the population.

To determine what this estimate means, one can naïvely match these cumulative percentages with self-reported ideology in surveys by assuming that these two represent the same population. Colors in Fig 7 gives the result by matching these data with General Social Surveys [12]. In the 2016 General Social Surveys, self reported ideologies from extreme liberal to extreme conservatives are: 4.9%, 12.7%, 11.2%, 37.4% (moderate), 13.9%, 15.5%, and 4.4%, respectively. This is quite close to Gallup’s 25% liberal, 34% moderate, and 36% conservative estimate [25]. The latest numbers in National Election Study we can find is in year 2012 [13].

**Fig 8:**
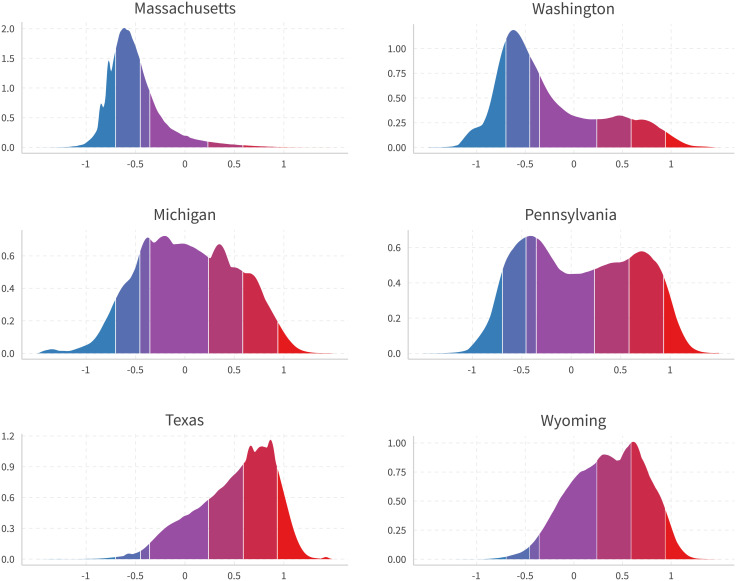
Users in selected liberal, swing, and conservative states. States are guessed by the maximum state on likes of national politicians (Sen, Rep, Gov). Colors represent matching densities with self-reported ideology shares in 2016 General Social Surveys [12].

**Fig 9:**
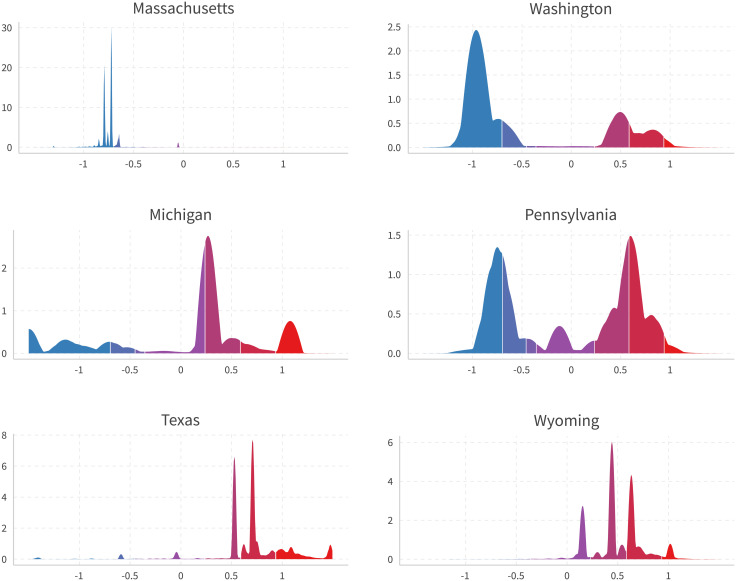
Users in selected states, politician-only method [1]. This figure shows user ideology estimate using only politician pages [1]. States are guessed by the maximum state on likes of national politicians (Sen, Rep, Gov). Colors represent matching densities with self-reported ideology shares in 2016 General Social Surveys [12].

Fig 8 provides state level densities in six selected states. The top panel is consistently liberal states, the middle states swing from supporting Obama in 2012 to Trump in 2016, and the bottom is conservative states. Colors indicate quantiles matched to all U.S. users with national-level self-reported ideologies in General Social Surveys, the same as in Fig 7. We can observe disparities among ideology distributions between these states. Additionally, if we use only politician pages to calculate users’ ideology (the method used in [1]), we will get Fig 9. One can see sharp distinctions between the results of the two methods. Plots for all 50 states are presented in S7 Fig.

S6 Fig compares the overall results for users with the result using only politician pages [1]. The politician-only method not only indicates a more heavy-tailed distribution of users, it is also more jumpy and noncontinuous, which seems less consistent with the belief that there could be a more continuous and smooth representation of political beliefs across the population.

## Election prediction using facebook

### Ideology dynamics

One strength of our measure compared with other pre-existing social-media-based measures is that it may change over time (since we focus on the liking of posts).

**Fig 10:**
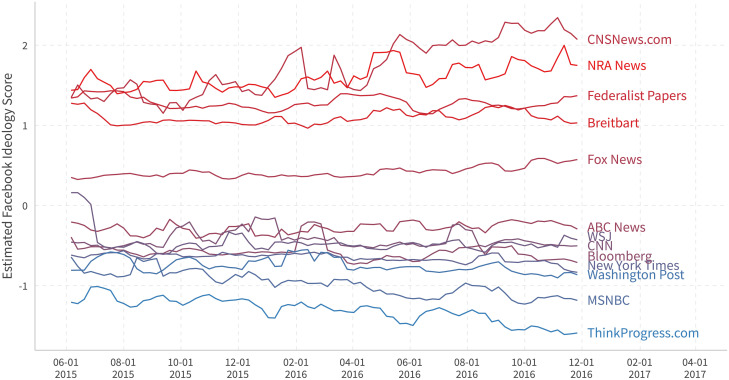
Ideological time series for selected news outlets. We use the data inside a moving window of one month (last 28 days) that updates every week to capture the dynamics of pages.

Fig 10 plots the time series for some news outlets, using data with a moving window of one month (last 28 days) that updates every week. One can observe that some pages, such as ABC News, the Wall Street Journal, and Fox News, are quite stable. Others, such as MSNBC and NRA News, tend to become more extreme as the election approaches.

A large number of rational choice theories are based on spatial models where political elites move to occupy a dense ideological spectrum and voters vote accordingly [4, 26]. Traditionally we can only test this in elections using vote shares, but since elections are rare, it is hard for us to observe any potential dynamic interactions.

**Fig 11:**
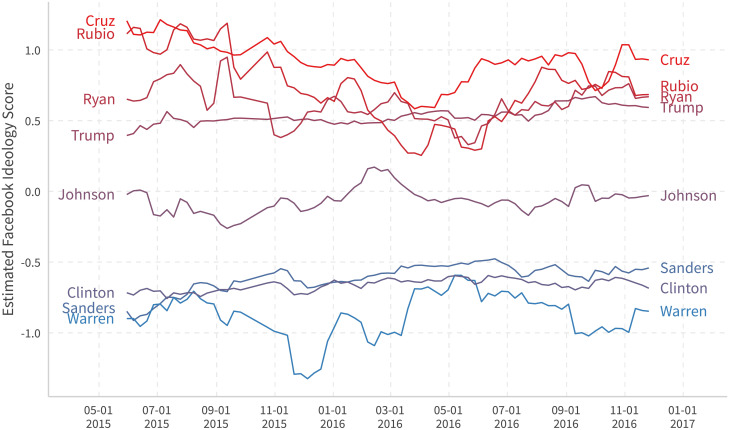
Ideological time series for major 2016 US politicians. We use the data inside a moving window of one month (last 28 days) that updates every week to capture the dynamics of pages. Marco Rubio withdrew on March 15th. Ted Cruz withdrew on May 3rd.

Fig 11 plots the ideological time series for the 2016 major Presidential primary candidates. It appears that most candidates tend to cluster around their own party centers during official primaries from February to June 2016. The estimates for Marco Rubio and Ted Cruz return to more extreme positions after their dropping out of the presidential race in March and May 2016, respectively.

### Forecasting 2016 presidential election

Another possible use of our data is to forecast elections. As a direct application of spatial models, we assume that people vote for candidates closer to their own ideological position, and given that we can guess the state that the user lives in, we can thus calculate the share of users closer to each candidate in each state. Although there may still be bias because turnout would not be uniform across states, and some other factors may also affect voting decisions, this can still be a reasonable forecast for election outcomes.

**Fig 12:**
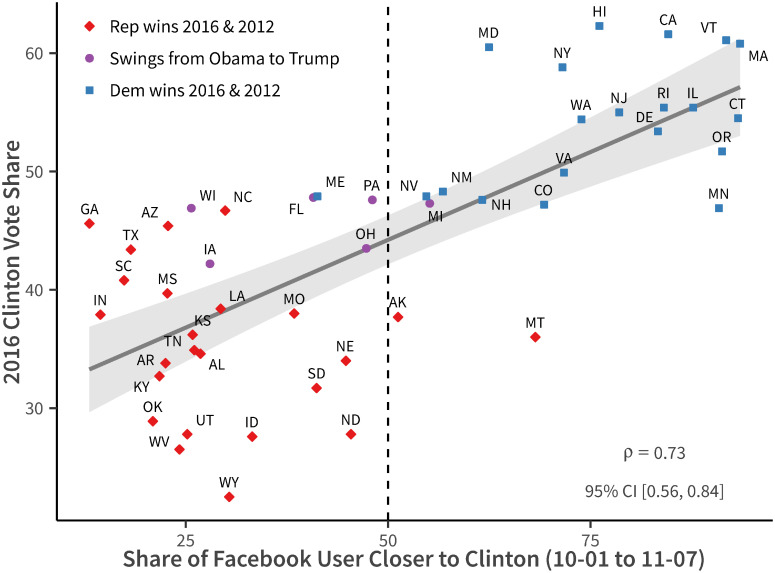
Forecasting 2016 presidential election. States are guessed by the maximum state on likes of national politicians (Sen, Rep, Gov). Data used: 2016-10-01 to 2016-11-07.

Fig 12 shows the result using data from between 2016-10-01 and 2016-11-07 (the election is held on 2016-11-08). On the x-axis we plot the share of users closer to Hillary Clinton in each state, and on the y-axis we plot the ex-post vote share Hillary Clinton gets. We can see that, relying only on ideology estimates, Facebook data predicts vote share quite well (*ρ* = 0.73).

Additionally, if we assume that Hillary Clinton wins those states where she is closer to more than 50% of users in term of ideology and Donald Trump wins the other states, we can almost get the national election outcome (with some exceptions in some truly small states such as Maine, Montana, and Alaska), where Trump gets a total of 292 out of 538 electoral college votes.

Table 4 compares our results with major election forecasts such as [27, 28], and the [29]. Our result is the most pessimistic for Hillary Clinton and the only one that correctly predicts the rise of President Trump. Furthermore, for most swing states, where the voters swing from Obama to Trump, we correctly predict the winner, excepting only Michigan.

**Table 4:** Election forecasts comparison.

State	Electoral Votes	Actual Winner	Facebook	538	NYT	PEC
Wisconsin	10	Trump	∘	×	×	×
Iowa	6	Trump	∘	∘	∘	∘
Florida	29	Trump	∘	×	×	×
Pennsylvania	20	Trump	∘	×	×	×
Ohio	18	Trump	∘	∘	∘	∘
Michigan	16	Trump	×	×	×	×
Maine	2	Clinton	×	∘	∘	∘
Alaska	3	Clinton	×	∘	∘	∘
Montana	3	Trump	×	∘	∘	∘
Trump’s Electoral Vote		306	292	235	216	215

*Notes*: Here we only list the states where Facebook estimate are wrong or generate different result with other forecasts. Wisconsin, Iowa, Florida, Pennsylvania, Ohio, and Michigan are states swings from Obama to Trump. *Sources*: [27–29]

### Similar trends between Facebook support rate and polling

One may also be curious about whether the time trend of Facebook support rates (percentage of users closer to that candidate in that state) is related to polling.

To compare Facebook support rates with polling, we scrape all the supporting rates from “Nowcast” (instead of forecasting the support rate on the day of election) on [27]. What FiveThirtyEight and other traditional forecasters do is collect several state-level and national-level polls, and then conduct many adjustments to get an average to project vote shares over time and across states.

**Fig 13:**
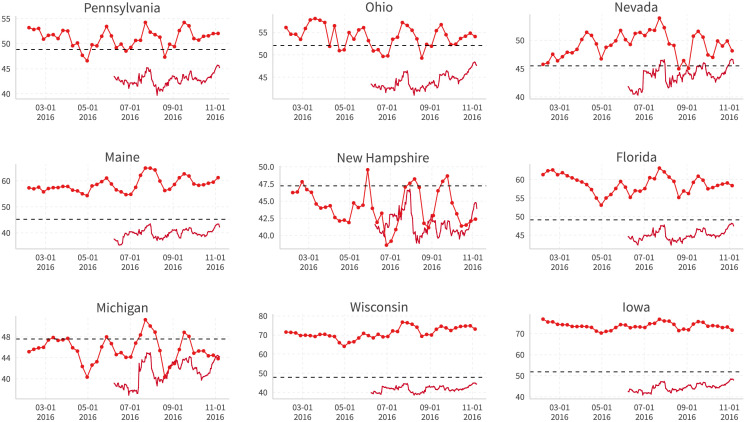
Trump’s Facebook support rate (dotted line), 538 polling average (undotted line), and 2016 actual vote share (dashed line) in selected battleground states. Facebook support rate: Share of user’s ideology closer to Trump. 538 Polling Average: “Nowcast” from [27]. Dickey-Fuller and Phillips-Perron cointegration tests after controlling for candidate-state fixed effects are implemented, where the null hypothesis of no cointegration between these two series is rejected.

Fig 13 shows the calculated Facebook support rate for Trump (dotted line), the polling average (undotted line, taken directly from the “nowcast” on [27]), and the actual vote share on election day (dashed line) for some battleground states. The results for all 50 states are presented in S8 Fig (Trump) and S9 Fig (Clinton).

Although this support rate measure is simple and we don’t make any further adjustments or add other assumptions (such as those in polling averages), we find that in many states, especially where voters tend to be in a 50–50 split, the similarities between the trends in Facebook support rates and polling averages are quite high.

**Fig 14:**
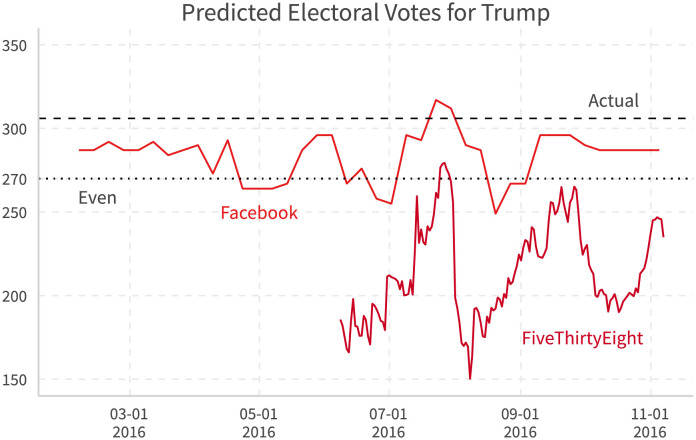
Expected electoral votes based on Facebook and polling. Facebook: Trump wins a state if the share of user’s ideology closer to Trump is higher. 538: “Nowcast” from [27].

After calculating the support rates over time, we can derive the estimated time series for the electoral college votes for Trump. [27]also provides its prediction of the total electoral votes for each candidates over time. Fig 14 compares our predicted electoral votes for Trump with those on [27]. We find that not only are the trends quite similar between Facebook and polling-based FiveThirtyEight estimates, Facebook estimates are closer to the actual electoral votes most of the time.

To test whether there exists a statistically significant connection between Facebook state support rates and state-level polls, we employ the standard Dickey-Fuller and Phillips-Perron cointegration tests under a panel data setting which controls for candidate-state pair fixed effects. The null hypothesis of no cointegration between these two series is rejected (p-value for Dickey-Fuller t, Modified Dickey-Fuller t, Augmented Dickey-Fuller t, and Phillips-Perron t tests = 0.0000; p-value for Modified Phillips-Perron t test = 0.0343).

**Fig 15:**
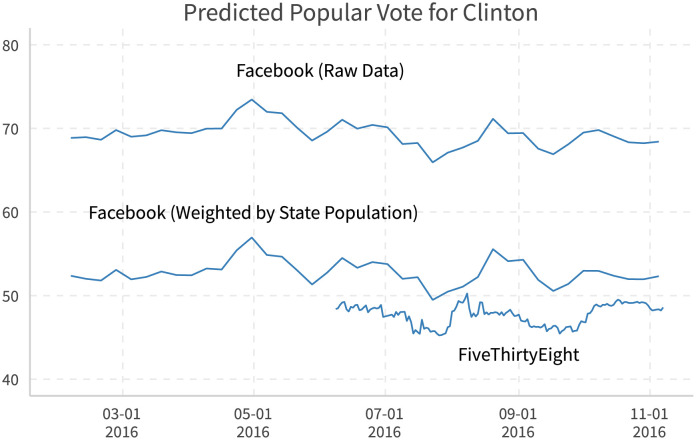
Forecasting popular vote for clinton. Facebook (Raw Data): Calculated using all US political users that have closer ideological positions to Clinton. Facebook (Weighted by State Population): Calculated using the population in each state from [23], multiplied by Facebook support rates in each states. FiveThirtyEight: “Nowcast” from [27]. States are guessed by the maximum state on likes of national politicians (Sen, Rep, Gov). Data used: 2016-10-01 to 2016-11-07.

Not only do state-wide and electoral vote forecasting generate similar results with those from polling, the popular vote forecasting also performs quite well. Fig 15 plots the time series for Clinton popular votes using Facebook raw data, Facebook data weighted by state population, and popular vote data provided by [27]. Since Facebook users may not be representative, the popular vote calculated using raw data is far from accurate. However, after minor adjustments (weighted by state population from [23]), the popular vote prediction becomes much more reasonable. It also shares similar trends with the polling-based result from [27].

### Difference between Facebook support rate and polling

**Fig 16:**
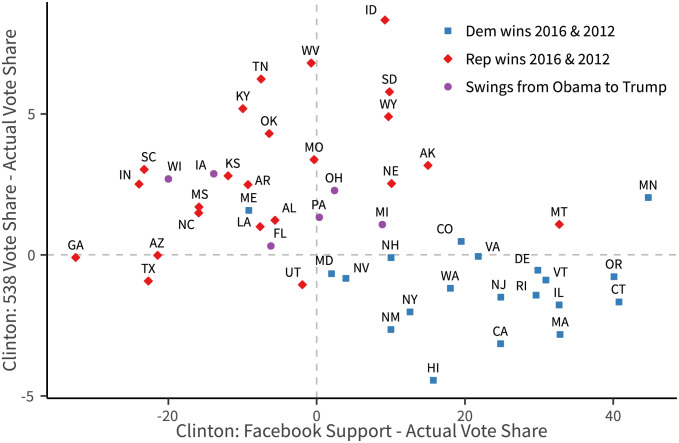
Compare clinton’s Facebook support rate and polling-based predicted vote share with actual vote share. Facebook support rate: Share of user’s ideology closer to Clinton, using data one month before the week of election (2016-10-09 to 2016-11-05). 538 Polling Average: “Nowcast” for 2016-11-08 from [27].

We also find some systematic differences between the Facebook-based measure and polling-based measure. Fig 16 shows the difference between Clinton’s Facebook support rate and Clinton’s actual vote share (x-axis) and the difference between Clinton’s polling average (taken from [27]) and Clinton’s actual vote share (y-axis) for each state.

Overall, polling averages, compared to the Facebook support rate, are closer to the actual vote shares. However, above the horizontal zero line–where polling averages overestimate Clinton’s performance–are mostly comprised of red and swing states. Additionally, most red and swing states lie in this region, which may suggest that polling averages tended to systematically overestimate Clinton’s support rate in these right-leaning states.

**Fig 17:**
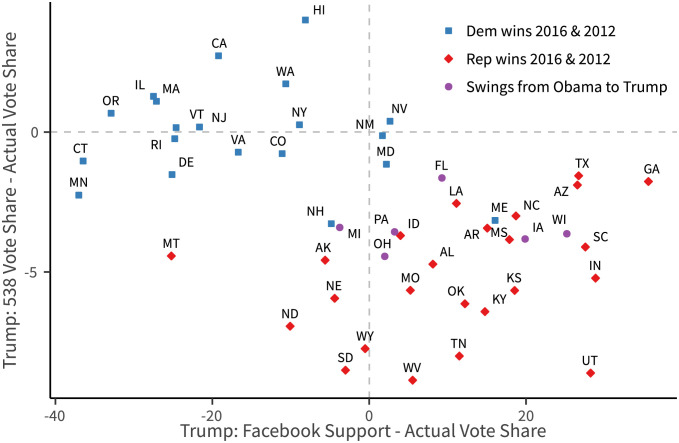
Compare Trump’s Facebook support rate and polling-based predicted vote share with actual vote share. Facebook support rate: Share of user’s ideology closer to Clinton, using data one month before the week of election (2016-10-09 to 2016-11-05). 538 Polling Average: “Nowcast” for 2016-11-08 from [27].

On the other hand, this systematic bias is considerably less prominent in Facebook estimates, while often the opposite is true on Facebook—Trump’s support rates are often overestimated in red and swing states—as illustrated in Fig 17.

**Fig 18:**
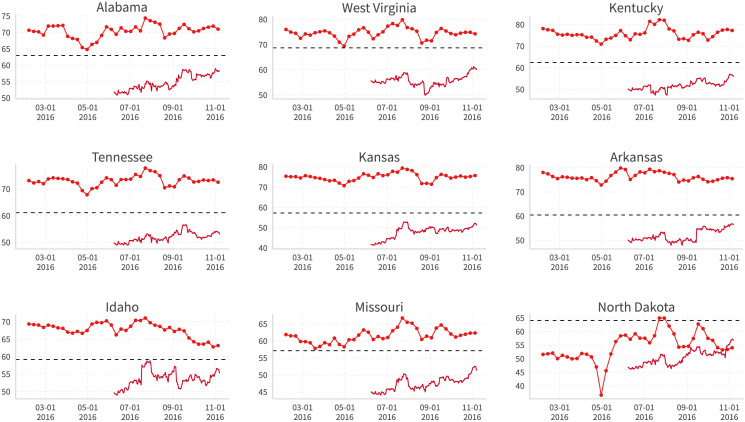
Trump’s Facebook support rate (dotted line), 538 polling average (undotted line), and 2016 actual vote share (dashed line) in selected red states. Facebook support rate: Share of user’s ideology closer to Trump. 538 Polling Average: “Nowcast” from [27]. Dickey-Fuller and Phillips-Perron cointegration tests after controlling for candidate-state fixed effects are implemented, where the null hypothesis of no cointegration between these two series is rejected.

**Fig 19:**
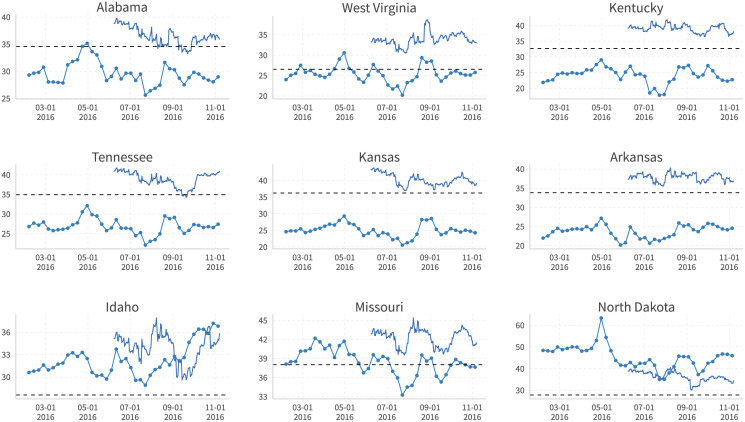
Clinton’s Facebook support rate (dotted line), 538 polling average (undotted line), and 2016 actual vote share (dashed line) in selected red states. Facebook support rate: Share of user’s ideology closer to Clinton. 538 Polling Average: “Nowcast” from [27]. Dickey-Fuller and Phillips-Perron cointegration tests after controlling for candidate-state fixed effects are implemented, where the null hypothesis of no cointegration between these two series is rejected.

Moreover, this systematic bias is not correlated with time or the approach of the election. Figs 18 and 19 compare the Facebook support rate for Trump/Clinton (dotted line), the polling average (undotted line, taken directly from the “nowcast” on [27]), and the actual vote share on election day (dashed ine) for some right-leaning states. We find that although the trends are quite similar between Facebook estimates and polling-based support rates, Facebook-based estimates almost always overestimate Trump’s/underestimate Clinton’s support, while polling-based estimates overestimate Clinton’s/underestimate Trump’s performance quite often.

What are the reasons for this difference? First, we have to make clear that the support rate we calculated is not a direct estimator of vote shares. Vote share not only depends on the actual support for a candidate, it also depends on turnout. Conservative voters in red states, despite being strong supporters of Trump, may have much less incentive to vote. This may explain why support rates derived from Facebook can be much higher than actual vote shares.

However, this still cannot explain why polling averages often underestimate Trump’s performance, since they usually have already taken the intent to vote into account.

Another possibility is that Trump supporters are more willing to show their preference on social media (this could be enhanced by their network), while fewer people are willing to do so with pollsters. Nevertheless, we do not have any direct evidence to test this hypothesis.

## Discussions and conclusions

In this paper, we use Facebook data to measure ideology—not only for politicians but also for users and news outlets. We then use this measure to derive support rates for candidates, to predict the outcome of elections and to compare them with polling averages. We find that under very minimal but intuitive assumptions, our ideology measure and Facebook support rate performs quite well relative to traditional polling measures and actual election results.

To our knowledge, this is the only attempt to make election predictions by combining state of the art scaling methods on social media and the canonical model from social science theory. There are many ways people can develop more complicated methods and incorporate more delicate theories, but we document here that classical social science theories and simple methods are already quite informative—without too much researcher judgment involved.

There are some particular strengths to our Facebook-based method. First, compared to surveys or polling, making inferences using social media data is inexpensive, can be undertaken almost in real time, and can trace individuals repeatedly over time. To conduct surveys or polls at the scale of social media data is not practical.

Additionally, our method is based on a revealed preference approach instead of a direct ask for self-disclosure. This may reduce the possible social desirability bias imposed by the respondent’s views of the pollsters or the surveyors, especially if the respondent thinks they may already have a certain view towards their answers. However, there can still be social desirability bias *among the users* on Facebook, since people may experience pressure from their peers to act in a certain way. This may be part of the explanation of our overestimate of Trump’s support rate in conservative states (and also Clinton’s support rate in liberal states).

There are also limitations to making inferences using Facebook data. Representativeness may be one of the most prominent problems, since we don’t truly know much about the users other than their behaviors on Facebook. It is very hard to address this problem without having a better understanding of Facebook users in general, or having access to more data other than their behavior on Facebook, such as some fundamental sociodemographic information.

Additionally, there is still little known about how to link behaviors online and offline. For example, one can derive the strength of a user’s supportive for a candidate compared to others from their online behaviors. This is usually difficult to show in polls. However, it is hard to know whether or how this would translate to important offline behaviors, such as voting.

It is important to note that these two methods are not substitutes. Social media data can be substantially enhanced by weighting to form a more representative sample. Survey and polling can likewise take behaviors on social media into account, and help to link online behaviors with offline behaviors. There is room for improvement in both techniques from benefiting from each other.

## Supporting information

S1 Fig Ideological density for newspaper pages. Data used: 2016-10-01 to 2016-11-24.
(EPS)

S2 Fig Ideological density for TV, radio, and website pages. Data used: 2016-10-01 to 2016-11-24.
(EPS)

S3 Fig Ideological density for public figure pages. Data used: 2016-10-01 to 2016-11-24.
(EPS)

S4 Fig Ideological density for party pages. Data used: 2016-10-01 to 2016-11-24.
(EPS)

S5 Fig DW-nominate vs. FB estimate (115 Congress). (EPS)

S6 Fig User density by politician-only [1] vs. our method. Blue region represents the method used in our paper. Red region uses the procedure suggested by [1] where one only considers politician fan pages and calculate user ideology accordingly. We remove a huge jump created by users only like one page: Arnold Schwarzenegger.
(EPS)

S7 Fig User densities by 50 states with national ideology shares in GSS. States are guessed by the maximum state on likes of national politicians (Sen, Rep, Gov). Colors represent matching densities with self-reported ideology shares in 2016 General Social Surveys [12]. We remove a huge jump created by users only like one page: Arnold Schwarzenegger.
(EPS)

S8 Fig Trump’s Facebook support rate (dotted line), 538 polling average (undotted line), and 2016 actual vote share (dashed line) in all 50 states. Facebook support rate: Share of user’s ideology closer to Trump. 538 Polling Average: “Nowcast” from [27]. Dickey-Fuller and Phillips-Perron cointegration tests after controlling for state fixed effects are implemented, where the null hypothesis of no cointegration between these two series is rejected.
(EPS)

S9 Fig Clinton’s Facebook support rate (dotted line), 538 polling average (undotted line), and 2016 actual vote share (dashed line) in all 50 states. Facebook support rate: Share of user’s ideology closer to Clinton. 538 Polling Average: “Nowcast” from [27]. Dickey-Fuller and Phillips-Perron cointegration tests after controlling for state fixed effects are implemented, where the null hypothesis of no cointegration between these two series is rejected.
(EPS)
